# Does acupuncture have a role in the treatment of threatened miscarriage? Findings from a feasibility randomised trial and semi-structured participant interviews

**DOI:** 10.1186/s12884-016-1092-8

**Published:** 2016-10-07

**Authors:** Debra Betts, Caroline A. Smith, Hannah G. Dahlen

**Affiliations:** 1 National Institute of Complementary Medicine, Western Sydney University, Locked Bag 1797, Penrith, NSW 2751 Australia; 2 School of Nursing and Midwifery, Western Sydney University, Locked Bag 1797, Penrith, NSW 2751 Australia; 3 New Zealand School Acupuncture and Traditional Chinese Medicine, P.O. Box 11076, Wellington, 6142 New Zealand

**Keywords:** Threatened miscarriage, Randomised controlled trial, Feasibility study, Mixed methods, Acupuncture, Supportive care

## Abstract

**Background:**

Threatened miscarriage is a common complication of early pregnancy increasing the risk of miscarriage or premature labour. Currently there is limited evidence to recommend any biomedical pharmacological or self-care management, resulting in a ‘watchful waiting’ approach. The objective of this study was to examine the feasibility of offering acupuncture as a therapeutic treatment for women presenting with threatened miscarriage.

**Methods:**

A mixed methods study involving a randomised controlled trial and semi structured interviews. A pragmatic acupuncture protocol including medical self-care advice was compared to an active control receiving touch intervention and medical self-care advice. Descriptive statistics were used to examine the demographic and baseline characteristics. Endpoints were analysed between groups using a mean *t*-test and chi-square tests with *P* < 0.05 considered statistically significant. Dichotomous data was expressed as Risk Ratio with 95 % confidence intervals. Eleven participants were purposively interviewed about their experiences on exiting the trial with interviews analysed using thematic analysis.

**Results:**

Forty women were successfully randomised. For women receiving acupuncture there was a statically significant reduction with threatened miscarriage symptoms including bleeding, cramping and back pain compared with the control (*p* = 0.04). Thematic analysis revealed women were dissatisfied with the medical support and advice received. An overarching theme emerged from the data of ‘finding something you can do.’ This encompassed the themes: ‘they said there was nothing they could do,’ ‘feeling the benefits’ and ‘managing while marking time.’

**Conclusion:**

Acupuncture was a feasible intervention and reduced threatened miscarriage symptoms when compared to a touch intervention. Further research is required to further explore acupuncture use for this common complication and whether it can reduce the incidence of miscarriage.

**Trial registration:**

Australian New Zealand Clinical Trials Registry (ANZCTR), ACTRN12610000850077. Date of registration 12/10/2010. Retrospectively registered, with first participant enrolled 11/10/2012.

**Electronic supplementary material:**

The online version of this article (doi:10.1186/s12884-016-1092-8) contains supplementary material, which is available to authorized users.

## Background

Vaginal bleeding is reported as a common complication in early pregnancy affecting an estimated 20 % of pregnant women [16]. Threatened miscarriage involves vaginal bleeding with a confirmed viable pregnancy [38]. These pregnancies may remain at risk; with twice the rate of miscarriage when bleeding is light and four times with heavy bleeding [41]. Threatened miscarriage is also associated with complications such as antepartum haemorrhage (APH) and premature delivery [34, 39].

Evidence to date fails to support biomedical medications or lifestyle interventions such as bedrest to improve birthing outcomes [1, 14, 15, 40], resulting in a medical monitoring approach of “watchful waiting.” While it is known that chromosomal abnormalities are responsible for approximately 50 % of miscarriages [17], the cause for the majority of the remaining miscarriages remains unknown [10]. With threatened miscarriage it is possible the bleeding has specific causes such as subchorionic hematomas or early pregnancy hormonal responses that if resolved, would improve pregnancy outcomes.

Traditional acupuncture has specific theories relating to promoting optimal early pregnancy responses and is recommended as a treatment modality for threatened miscarriage in acupuncture texts [4, 24, 26, 42]. The potential for acupuncture as a therapeutic treatment for threatened miscarriage has been outlined in early pregnancy [5]. This includes assisting women’s response to stress in early pregnancy and providing supportive care, which has been shown to improve pregnancy rates for women with unexplained recurrent miscarriage. Although it is known that acupuncturists treat threatened miscarriage [7, 35], there are no quality trials to examine the safety or use of this treatment [5]. With a growing interest from women seeking acupuncture during pregnancy [2, 36] and a willingness from biomedical health practitioners to refer pregnant women [37], acupuncture may provide an alternative treatment option to medical ‘watchful waiting.’ The aim of this study was to examine the role of acupuncture as a therapeutic treatment for women presenting with threatened miscarriage.

## Methods

### Study design

The objectives of this study were twofold – firstly to examine the feasibility and acceptability of offering acupuncture as measured by recruitment and retention. A second objective was to explore clinical outcomes with ongoing pregnancy as a primary outcome and physical and emotional wellbeing as secondary outcomes. This was through a mixed methods study involving a pragmatic randomised controlled trial and semi structured interviews on the women’s experiences on exiting the trial. For women experiencing threatened miscarriage, trial allocation was to a group receiving acupuncture and individualised self-care advice or an active control group receiving a touch intervention receiving medical self-care advice. The randomisation sequence was computer-generated and managed by Sealed Envelope, an Internet randomisation service. Allocation was held centrally and accessed by computer by the researcher (DB).

All women received two visits for the first week of intervention, followed by a weekly visit until 12 completed weeks of gestation. Treatments were delivered in the setting of an acupuncture school clinic or if requested in the women’s home environment. The acupuncture treatment protocol was based on a choice of an eight principle diagnosis from textbook recommendations [4, 24, 26, 42], with additional flexibility to treat underlying patterns of disharmony, historical diagnostic approaches that are still in use today among traditional acupuncture practitioners, according to an established acupuncture text [25]. The needles brand used was ‘Hwato’. Single use disposal needles: 0.22 × 25 mm, 0.25 × 40 mm and 0.30 × 75mm The acupuncture intervention included: needles, moxibustion therapy with smokeless moxa sticks (a therapy that involving providing heat to acupuncture points), and self-care advice specific to the diagnosis made by the researcher (DB), a licensed practitioner with over 20 years clinical experience in delivering pregnancy acupuncture. This self-care advice related to the women’s individual diagnosis and contained explanations that related to traditional Chinese medicine theory (see Additional file 1). Point location and needling depths were according to a recognised acupuncture text [13]. A manual needling technique was used for obtaining deqi (a characteristic needling sensation sought when treating by traditional acupuncturists), with a needle retention time of 20 min, with a maximum of six acupuncture points at any one treatment.

Usual care is recommended as a control for use in pragmatic trials, however, with no usual care other than “watchful waiting’ there were ethical concerns that randomising women to a no-treatment group was unacceptable. Therefore an active control group received touch to non-acupuncture points and medical self-care advice (see Additional file 2). This offered components to control for the non-specific therapeutic effects offered by acupuncture, such as receiving attention, touch, time out to rest and having a health practitioner to consult. This light touch was provided by the researcher (DB). Light touch was used as this has been successfully used in acupressure trials during childbirth to control for these non-specific therapeutic effects [11, 19, 21, 22].

### Participants

Participants were women with vaginal bleeding and a viable pregnancy of 6–11 completed gestational weeks, living in the Wellington and Hutt Valley areas of New Zealand. Light vaginal bleeding was defined as less than the flow of a menstrual period and lasting longer than a day, with heavy bleeding defined as bleeding heavier than the flow of a menstrual period. Recruitment was undertaken through health professionals providing women with an information pamphlet. Practitioners referred through a fertility unit, a Maternity Assessment Unit (MAU) within a hospital and midwives practicing as lead maternity caregivers in the community.

### Data collection and statistical methods

Data to assess the objective of feasibility was collected for recruitment rates, referral sources, eligibility criteria, and retention rates. Data for ongoing pregnancy rates was collected through telephone follow up and hospital records at 12 and 20 gestational weeks and at birth for pregnancy loss and pregnancy and perinatal outcomes.

Data to assess physical and emotional wellbeing was collected at baseline then weekly until women exited the trial. Wellbeing was assessed using the Measure Yourself Medical Outcome Profile (MYMOP) questionnaire; a validated questionnaire used to assess patient centred outcomes in Complementary and Alternative medicine (CAM) research [30–32]. Participants were asked to score their general wellbeing and two symptoms they nominated as a concern on a continuum between 0 and 6, with 6 being as bad as they could imagine.

No previous randomised trials have evaluated the potential of acupuncture on pregnancy outcomes for women presenting with threatened miscarriage. In this pilot trial, a modest sample size of 40 women was sought to provide data to answer the study questions. This number of participants allowed for the examination of the study questions relating to feasibility.

Data was entered into an Excel spreadsheet and exported to SPSS version 19.1 for analysis. Analysis was undertaken blind to group allocation. Descriptive statistics were used to examine the demographic and baseline characteristics of trial participants. Analyses of the endpoints used an ‘intention to treat’ approach and compared differences in the outcomes of the groups. Endpoints were analysed between groups using a mean *t*-test and chi-square tests with *P* < 0.05 considered statistically significant. Dichotomous data was expressed as Risk Ratio with 95 % confidence intervals.

#### Interviews with women

Semi structured interviews were conducted with women in the order they exited the trial by a researcher with no involvement in delivering the interventions. Eleven women were interviewed before it was determined that no new information was produced in analysis and therefore saturation had been reached [18]. A topic guide consisting of five questions asked participants to share their experiences of receiving acupuncture or touch (see Additional file 3). Interviews typically lasted less than an hour, were recorded and then stored on the researcher’s (DB) computer. Data was analysed using thematic analysis. This involved the researcher (DB) becoming familiar with the data, generating initial codes, searching for themes, reviewing, defining and then naming these themes [9]. To reduce possible researcher bias NVivo 9 was used. This allowed coding categories to be sent to the second and third author for comparison and verification. The authors (HD & CS) provided critical feedback and reflection as themes developed.

## Results

### Feasibility outcomes

#### Recruitment and participant flow

**Fig. 1:**
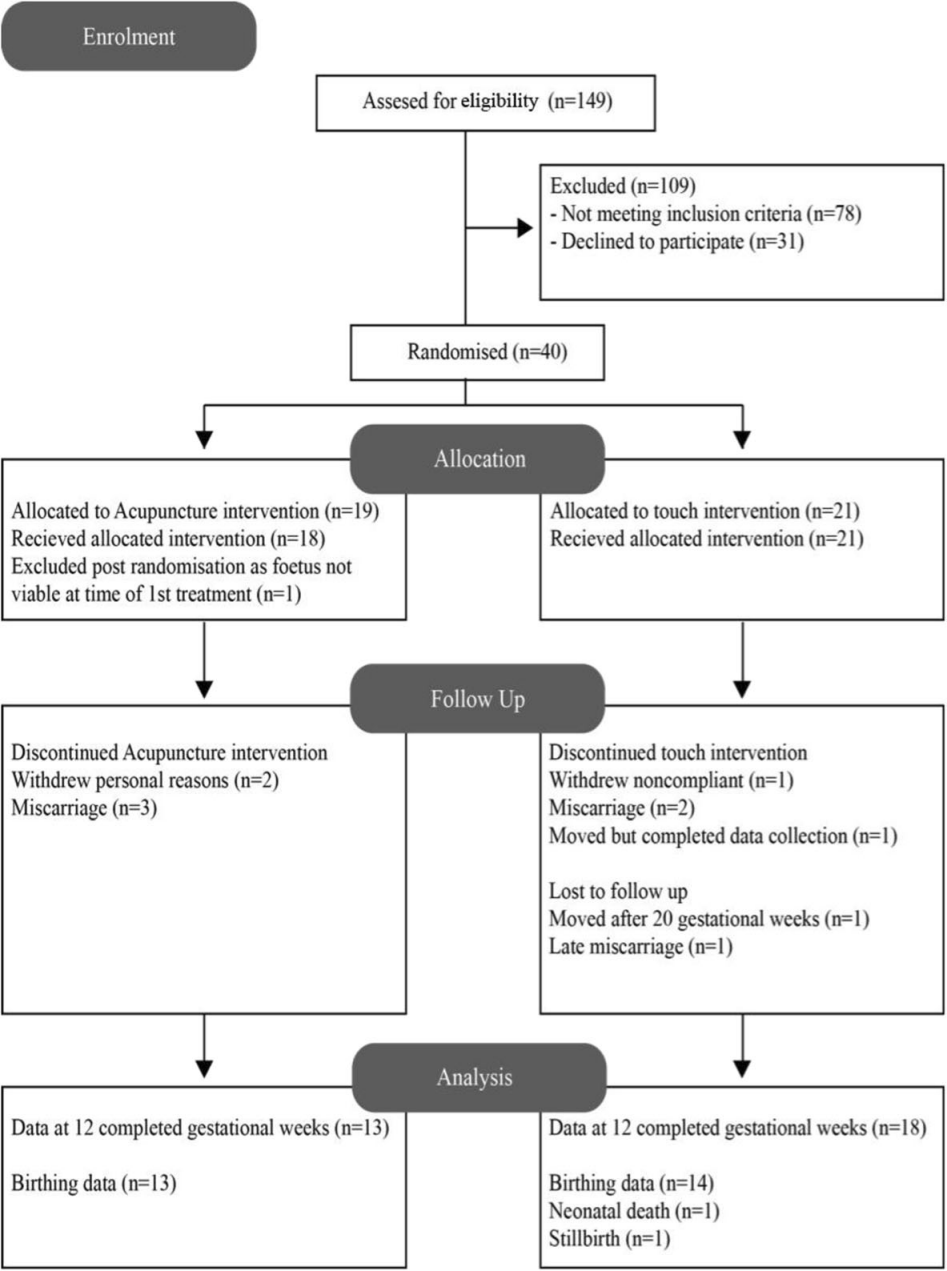
Flow diagram of participants in study

On assessment for eligibility half (78, 52.3 %) of the 149 women initially referred did not meet the entry requirements (Fig. 1). The majority of those not meeting the entry criteria (44, 56.4 %) presented at 11 plus gestational weeks. The primary source for women successfully referred occurred through a MAU (17, 42.5 %). One woman was inappropriately randomised into the trial due to human error – failure to determine if her ultrasound scan was recent which resulted in her presenting for treatment with a nonviable pregnancy. She was therefore excluded post randomisation.

### Acupuncture treatment considerations: acceptability and diagnosis

Women from both groups received an average of four treatment sessions suggesting that women found the acupuncture intervention acceptable (mean of 4.1 (SD 1.6) for the touch group and 4.3 (SD 1.8) for the acupuncture group). From 31 possible TCM diagnoses, 12 were utilised (Table 1). A total of 28 acupuncture points were used (Table 2) with moxibustion also used between visits for three women (16.6 %). No major adverse events occurred.

**Table 1:** TCM patterns of disharmony used in diagnosis for threatened miscarriage

TCM patterns of disharmony (*n* = 12)	Number	Percent
Constrained Liver qi	9	75.0
Liver blood deficiency	4	33.3
Spleen yang deficiency	4	33.3
Qi deficiency with blood stasis	4	33.3
Blood stasis	3	25.0
Kidney yang deficiency	3	25.0
Spleen failing to control blood	3	25.0
Restless Heart qi	3	25.0
Kidney qi deficiency	2	16.6
Kidney yin deficiency	1	8.3
Spleen deficiency with dampness accumulation	1	8.3
Trauma	1	8.3

**Table 2:** Acupuncture points used in treatment

Meridian	Acupuncture points
Lung	LU 7
Large intestine	LI 11
Heart	HT 7
Liver	LR 2, LR 3
Gall bladder	GB 30, GB 34, GB 41
Spleen	SP 1, SP 4
Stomach	ST 36, ST 37
Kidney	KI 6, KI 9, KI 21, KI 27
Bladder	BL 20, BL 23, BL 57, BL 62
Pericardium	PC 6
Triple energiser	TE 4, TE 5, TE 6
Conception vessel	CV 4
Governing vessel	GV 4, GV 20
Non-meridian	Yin Tang

### Baseline characteristics

Most participants were in a relationship and had previously used CAM (Table 3). Mean maternal age was 30.5 years (SD 5.9). The mean gestational age at which vaginal bleeding commenced was 6.2 weeks (SD 1.8) with the mean gestational age for entering the trial 8.3 weeks (SD 1.4). A total of nine women were undergoing fertility treatment at the time of trial entry. There were no significant group differences detected between any baseline variables at trial entry.

**Table 3:** Characteristics of participants and MYMOP questionnaire at trial entry

Variables		Touch (*n* = 21)		Acupuncture (*n* = 18)	
		Mean	SD	Mean	SD
Maternal age		30.6	7.2	30.4	4.3
Gestational age of bleeding		6.5	1.8	5.9	1.8
Gestational at trial entry		8.2	1.3	8.3	1.6
		n	%	n	%
Previous miscarriage		10	47.6	6	33.6
Fertility patient		6	28.6	3	16.7
Bleeding	Heavy	6	28.6	4	22.2
	Light	15	71.4	14	77.8
Employment	Full time/part time	14	66.7	13	72.2
	Unemployed	2	9.5	1	5.6
	Home duties	3	14.3	1	5.6
	Student	2	9.5	3	16.7
Education	High school	16	76.2	15	83.3
	Polytechnic	6	28.6	3	16.7
	University	9	42.9	10	55.6
Race	Caucasian	11	52.4	13	72.2
	Maori	5	23.8	2	11.1
	Other	5	23.8	3	16.7
Relationship	Single	2	9.5	4	22.2
	Married/de facto	19	90.5	14	77.8
Current CAM use		8	38.1	3	16.7
Past CAM use		19	90.5	17	94.4
Past Acupuncture use		7	33.3	8	44.4
MYMOP Questionnaire		Mean	SD	Mean	SD
		(*n* = 20)		(*n* = 18)	
General well being		2.9	1.5	3.3	1.4
		(*n* = 20)		(*n* = 17)	
Symptom 1		4.6	1.2	4.7	1.1
		(*n* = 7)		(*n* = 10)	
Symptom 2		2.8	0.9	3.9	0.9

*SD* Standard Deviation

*CI* Confidence interval

### Pregnancy outcomes

There were a total of eight pregnancy losses (Table 4). Although five women experienced pregnancy loss in the touch group compared to three women in the acupuncture group, this was not statistically significant (RR 0.70; 95 % CI, 0.19, 2.13, *p* = 0.58). There were no differences in pregnancy complications between groups for either stillbirth or neonatal death. While it was not possible to examine miscarriage tissue for chromosomal abnormality, the ultrasound report for one woman in the acupuncture group with early miscarriage did indicate chromosomal abnormality. For late pregnancy losses, one was unexplained, one followed an APH at 27 weeks and one woman had premature labour at 23 weeks. There were no differences in pregnancy complications between groups.

**Table 4:** Secondary outcomes by study group

	Touch Mean (SD)		Acupuncture Mean (SD)		Mean difference	95 % CI	*P* Value
MMOP Wellbeing score	(*n* = 17)		(*n* = 13)				
	2.5 (1.4)		2.3 (1.1)		0.22	(-0.75, 1.19)	0.65
	(*n* = 17)		(*n* = 12)				
Score 1	3.1 (1.7)		1.7 (1.6)		1.36	(0.07, 2.66)	0.04*
	(*n* = 6)		(*n* = 6)				
Score 2	2.3 (2.0)		2.6 (1.8)		-0.33	(-2.86, 2.19)	0.77
	Touch (*n* = 21)		Acupuncture (*n* = 18)		RR	95 % CI	*P* value
	n	%	n	%			
Miscarriage prior to 12 gestational weeks	2	9.5	3	16.7	1.75	(0.33, 9.34)	0.51
Late miscarriage prior to 20 gestational weeks	1	4.8	0	0.0	0.39	(0.02, 8.93)	0.55
Stillbirth	1	4.8	0	0.0	0.39	(0.02, 8.93)	0.55
Neonatal death	1	4.8	0	0.0	0.39	(0.02, 8.93)	0.55
Total pregnancy loss	5	23.8	3	16.7	0.70	(0.19, 2.53)	0.59
Premature birth before 34 gestational weeks	1	4.8	1	5.5	1.17	(0.08, 17.3)	0.91
Intrauterine growth restriction	1	4.8	0	0.0	0.39	(0.02, 8.93)	0.55
Antepartum haemorrhage	1	4.8	1	5.5	1.17	(0.08, 17.3)	0.91
Total pregnancy complications	3	14.2	2	11.1	0.78	(0.15, 4.15)	0.77

*P < 0.05

### Physical and emotional wellbeing outcomes

For the MYMOP questionnaire two women did not have any symptoms of concern and not all selected a second symptom of concern. The majority of the 54 symptoms selected related to emotional aspects such as anxiety over bleeding, and feelings of frustration and depression (24, 44.4 %), followed by abdominal cramping (13, 24.0 %) and general symptoms such as back pain (8, 14.8 %).

On exiting the study, women receiving acupuncture indicated less concern for their primary symptom (MD 1.36; 95 % CI, 0.07, 2.66, *p* = 0.04) compared with the control (Table 4). Whilst we were underpowered to demonstrate differences between groups we have preliminary findings suggesting some benefit to quality of life, however, these findings require caution due to the large confidence in intervals.

### Thematic analysis

Of the 11 women interviewed, seven had received the acupuncture treatment and four were from the control group. An overarching theme emerged from the data of ‘finding something you can do.’ This encompassed the themes: ‘they said there was nothing they could do,’ ‘feeling the benefits,’ and managing while marking time.

### Finding something you can do

This theme captures how women participated in the trial due to a perceived lack of control and how they welcomed the opportunity to participate in self-care in a positive way:

I actually felt like I was actively doing something to try and help my situation, because otherwise you are left in a little bit of a black hole (Shelly).

I think doing nothing feels quite helpless really. It’s all outside of your control anyway but at least trying acupuncture makes you feel like you’re actually doing something (Janice).

### They said there was nothing they could do

For all women, the initial advice they received from their health practitioners was difficult to accept. It was not that practitioners were viewed as neglectful or deliberately unkind—just that, as part of their job, they were treating threatened miscarriage as a common situation and failed to appreciate how distressing it was for women to be told to ‘wait and see; and ‘try and not worry’:

The fact that when you ring up somebody who’s meant to be there—the clinic who’s helped you conceive and they’re meant to be a bit more supportive and all they say is, there’s not much you can do about it. It’s a bit of a shock (Rachel).

I guess what really chimed with me was about being told to go home and try not to worry about it…and it’s impossible. You know it’s utterly impossible to try not to worry about it (Annie).

While women expected, and all received follow up ultrasound appointments, there was also an expectation they would receive advice and support from their practitioners. Women discussed their dissatisfaction in terms of staff not spending time to talk with them, the lack of follow up and a lack of information:

All that happened is every time I had a bleed I’d ring my doctor and I had to go in and have my bloods taken and go for a scan. There wasn’t really anything else. It was just you’ve got to wait and see (Shelly).

The doctors like, well there is nothing you can do. There’s no information given to me. I think I would be more proactive if someone said that to me now, I’d be like, well that’s actually not true. (Lucy).

### Feeling the benefits

This theme illustrates why women saw participation in the study as useful to them, including talking to someone who understood. This was seen as support that allowed them to express any guilt and negative feelings and also to reduce the pressure they felt they were placing on their partners and families:

Debra acknowledged how I was feeling …she was not my family. She wasn’t my friend. She was someone that knew about it (Kelly).

While the majority of women described both touch and acupuncture as beneficial in helping with their stress and anxiety, those women receiving acupuncture treatment also described beneficial physical effects relating to changes in bleeding, cramping and back pain that were not discussed by women in the touch group. For two women who had been experiencing heavy bleeding and abdominal cramping for several weeks, acupuncture was perceived as beneficial due to the symptom changes following their first treatment:

The next day my bleeding stopped…yeah, I was shocked and I was really happy. I was a little bit sceptical, and I kind of was thinking oh is it all just —this acupuncture stuff, is it all just in people’s heads kind of thing, but no. I know now that it works (Kelly).

I was going through quite a lot of cramping and I had a treatment and that went away straight away, which was brilliant (Jill).

### Managing while marking time

This theme illustrates how all women saw themselves as having to manage until they achieved the safety of their second trimester—that each week offered more hope and reassurance until they were able to ‘get to that 3 months and everything would be OK’ (Susie). Women sought reassurance through the presence of nausea, which was taken as a positive sign by all women who experienced it. Although ultrasounds were also spoken of as powerful reassurance, women did not necessarily see these as a guarantee that the pregnancy would continue with no further problems. Several women expressed concerns that there would be ongoing problems with their pregnancy:

Even after seeing the scan I kept thinking oh no, something’s going to go wrong. I’m not sure why (Ellen).

## Discussion

Acupuncture was a feasible intervention to offer women, with referral from biomedical health practitioners and women completing the treatment time for the trial. Half of the women approached to participate in the trial exceeded the gestational age limit for entry of 11 weeks. This was an unexpected finding and may require further consideration in future studies. It remains unknown if these women were presenting with vaginal bleeding that had initially commenced after 11 weeks, or if they had been only referred to MAU when bleeding continued or reoccurred at 11 weeks. There were women in the trial that commented they were initially reassured implantation bleeding was normal - they were only referred for further assessment when this bleeding continued. These preliminary findings that women may be presenting in the late first trimester period with threatened miscarriage warrants further exploration in future research to explore if there are benefits to offering women care in second trimester.

In assessing physical and emotional wellbeing this RCT demonstrated that for women receiving acupuncture, there was a statistically significant reduction in the threatened miscarriage symptom that they self-selected as a primary concern (*p* = 0.04). With these symptoms relating to women’s anxiety over vaginal bleeding as well as abdominal cramping and back pain. Due to the small number of women involved in this study and the resulting large confidence intervals these findings require cautious interpretation. However it was interesting that this finding was captured in the interviews, where while women discussed being proactive as reducing their anxiety, those receiving the acupuncture intervention also discussed the relief they felt from specific reductions in symptom in terms of abdominal bleeding, cramping and back pain.

It was interesting to note that, while women in the acupuncture group demonstrated a significant difference in the symptom of primary concern compared to the touch group, there was no corresponding significant difference for the outcomes of general wellbeing. It may be that even when women see improvements in their symptoms they remain concerned about pregnancy loss until they reach the perceived safety of the second trimester. Certainly the women interviewed discussed ‘marking time’ until they reached the safety of their second trimester; this perception of not being safe until this time may have influenced how women reported their general wellbeing throughout the study.

While more women receiving the touch intervention experienced pregnancy loss this was not statistically significant (*p* = 0.58), an expected finding as this study was not powered to demonstrate significant differences between groups. Based on this study a medium size effect is expected, and allowing for a 30 % loss, it is estimated that a total sample size of 170 women would be required to demonstrate significant differences between groups. There were two premature deliveries (5.1 %), and two women (5.1 %) with antepartum haemorrhage. This did not reflect previous research that women with threatened miscarriage remain at risk for pregnancy complications [12, 33, 34, 39].

It emerged from the semi-structured interviews that women entered the trial because they wanted to be proactive and were dissatisfied with the watchful waiting advice they received from their biomedical health practitioners. This dissatisfaction with medical advice that there is nothing women can do but ‘wait and see’ is also a finding of internet postings to threatened miscarriage forums [6]. It was also evident from the interviews that women were seeking support, including someone to talk to, and information from their health practitioners beyond regular ultrasound monitoring. This mirrors recurrent miscarriage research findings where ultrasound scans were less frequently requested by women than initially anticipated by the researchers and women requested support in terms of their physician listening and taking their concerns seriously [28, 29].

Biomedical research has focused on developing miscarriage prediction models for threatened miscarriage with the aim of improving the identification of those who are most likely to require further support when they miscarry [3, 8, 20]. No new research has provided a voice for women to articulate their needs for care. The findings that acupuncture may provide symptom relief for women experiencing threatened miscarriage provides new knowledge concerning how acupuncture could be used to assist women experiencing threatened miscarriage symptoms during their pregnancy. In addition interviews with women in this trial identified that women valued self-care advice and access to someone knowledgeable that they could talk to.

### Strengths and limitations of the study

The use of a mixed methods approach in this study identified preliminary findings that resulted in a greater understanding of how acupuncture supported women experiencing threatened miscarriage than possible from using a quantitative or qualitative approach in isolation. In addition the use of a pragmatic acupuncture treatment protocol allowed flexibility for diagnosis and treatment, increasing external clinical validity for practitioners by reflecting a treatment approach applicable to real-world acupuncture. Although pragmatic trial designs do not provide information on the effect of specific acupuncture points and needling effects, they are recommended when investigating acupuncture as a complex intervention. Where complex interventions refer to treatment effects not only derived from the use of needles, but also through dietary and lifestyle advice specifically tailored to meet diagnostic criteria that involve the participants [23, 27].

It was also a strength that the same researcher (DB) delivered both acupuncture and light touch intervention, allowing for consistency in the support given to both groups of women. The use of a sole practitioner to deliver the study interventions provided a consistent approach to the acupuncture diagnosis and treatment delivery, however, a sole practitioner delivering treatment also has the potential to introduce a treatment bias that may not be generalisable to other practitioners. Future pragmatic trials involving several acupuncturists would reduce this potential. A limitation was that it was not possible to blind women to treatment allocation and therefore not possible to control for patient expectation. The use of an active control group receiving touch was also a limitation as both groups received non-specific effects relating to supportive care. This may have impacted negatively on the findings for the acupuncture group however; it was not seen as ethical to randomise women to receive no care. To determine the effects of delivering acupuncture as a complex intervention treatment with effects not only derived from the use of needles, but also through dietary and lifestyle advice future research could focus on women receiving usual care rather than touch, for example in a population of women receiving fertility treatment with usual care in the form of ultrasound monitoring. The specific effects of needling such as acupuncture point selection, the depth, degree of stimulation, retention time and number of needles remain to be explored in future efficacy studies using a sham control.

## Conclusions

Acupuncture was a treatment viewed as acceptable for referral by health practitioners and considered by women to provide useful emotional support and relief from physical and emotional symptoms relating to threatened miscarriage. Acupuncturists may have an important role in working with health practitioners to offer care women perceive as relevant. Further research is justified to determine if the threatened miscarriage symptom changes found within the acupuncture group are generalisable and if this symptom reduction affects miscarriage rates.

## Additional files

Additional file 1: Acupuncture and Touch Protocol. This supplementary file describes the details of the acupuncture treatment and touch intervention delivered in this trial. (PDF 301 kb)
Additional file 2: Diet and Lifestyle sheet. This supplementary file details the medical self-care advice given to the women in this trial. (PDF 199 kb)
Additional file 3: Interview Schedule for Trial Participants. This supplementary file details the interview questions for women being interviewed. (PDF 260 kb)
