# Erratum to: Does acupuncture have a role in the treatment of threatened miscarriage? Findings from a feasibility randomised trial and semi-structured participant interviews

**DOI:** 10.1186/s12884-016-1206-3

**Published:** 2017-01-11

**Authors:** Debra Betts, Caroline A. Smith, Hannah G. Dahlen

**Affiliations:** 1 National Institute of Complementary Medicine, Western Sydney University, Locked Bag 1797, Penrith, NSW 2751 Australia; 2 School of Nursing and Midwifery, Western Sydney University, Locked Bag 1797, Penrith, NSW 2751 Australia; 3 New Zealand School Acupuncture and Traditional Chinese Medicine, P.O. Box 11076, Wellington, 6142 New Zealand

## Erratum

“After publication of our article [1] we realised that our Competing Interests statement should have read as follows: “Caroline Smith has had an association with The Acupuncture Pregnancy Clinic as the result of another unrelated research study. She states that she has not received any financial compensation for this relationship at any time.” ”
