# Supplementary material for: Does acupuncture have a role in the treatment of threatened miscarriage? Findings from a feasibility randomised trial and semi-structured participant interviews
Source: BMC Pregnancy Childbirth. 2016 Oct 7;16:298. doi: 10.1186/s12884-016-1092-8 (PMC5055689; doi:10.1186/s12884-016-1092-8)
Supplement: Additional file 1: — Acupuncture and Touch Protocol. This supplementary file describes the details of the acupuncture treatment and touch intervention delivered in this trial. (PDF 301 kb) [file 12884_2016_1092_MOESM1_ESM.pdf]

ACUPUNCTURE AND TOUCH PROTOCOL

The Role of Acupuncture Treatment with the Management of Threatened Miscarriage: An exploratory study

Acupuncture treatment

This involves receiving an acupuncture treatment that may include: needles, moxibustion therapy, dietary and lifestyle advice that is specific to the acupuncture diagnosis made by the acupuncturist.

Treatment consists of two treatments in the first week of entering the trial followed by a weekly visit until participants reach 12 completed weeks of gestation.

Treatment protocols include:

1. treatment based on an eight-principals TCM diagnosis
2. a selection of acupuncture points for each TCM diagnosis along with the appropriate indications for the use of moxibustion and/or cupping
3. recommendations for dietary and lifestyle advice that are specific for each TCM diagnosis
4. needling specifications for needling depths according to a recognised acupuncture text (Deadman, Al-Khafaji & Baker 2001)
5. needling, moxibustion and cupping techniques for early pregnancy according to recognised acupuncture obstetric texts (Betts 2006; Maciocia 1998; West 2001)
6. needling technique for obtaining deqi for each point and needle retention time of 20–30 minutes at each session
7. number of acupuncture points pre-treatment to be chosen by acupuncturist as required through acupuncture diagnosis, with the minimum number of acupuncture points being one and six being the maximum number of acupuncture points to be used in any one treatment.

All acupuncture treatment to be carried out in accordance with the ethical and skin penetration guidelines issued by the New Zealand Register of Acupuncture.

A written sheet detailing dietary and lifestyle risk factors is to be given to women receiving acupuncture care. These diet and lifestyle sheets are based on the TCM diagnosis women receive.

Acupuncture diagnosis

Diagnosis will include pattern differentiation based on a TCM paradigm and a treatment documented within a recognised acupuncture obstetric text (Betts 2006; Maciocia 1998; West 2001). Terminology of TCM terms and meridians based on WHO International Standard Terminologies (WHO 2007).

Lung Disharmonies

- Lung qi deficiency

LU 9, BL 13, BL 43

- Lung yin deficiency

LU 9, BL 13, BL 43

Kidney Disharmonies

- Kidney qi deficiency

KI 3, CV 4, GV 4, BL 23, GV 20

- Kidney yin deficiency

KI 3, CV 4, GV 4, BL 23, GV 20, CV 4

- Kidney yang deficiency

KI 7, CV 4, GV 4, BL 23, GV 20, CV 4

Spleen Disharmonies

- Spleen qi deficiency

GV 20, BL 20, BL 21, BL 43, CV 4

- Spleen yang deficiency

GV 20, BL 20, BL 43, CV 4

- Spleen failing to control the blood

GV 20, BL 17, BL 20, CV 4, SP 10

- Dampness damaging Spleen yang

SP 9, CV 12, BL 20, BL 21

- Spleen deficiency with dampness accumulation

SP 9, SP 9, CV 12, BL 20, BL 21

- Sunken Spleen qi

GV 20, BL 20, CV 4

- Spleen—Stomach weakness

BL 20, ST 36

Heart Disharmonies

- Heart qi deficiency

PC 6, HT 5, BL 15, CV 17, Yin Tang

- Restless Heart qi

PC 6, Yin Tang, GB 13, DU 24

- Heart yin deficiency

HT 7, PC 6, HT 6, KID 7, KI 6, CV 14, Yin Tang

- Heart yang deficiency

PC 6, HT 5, BL 15, CV 17

- Heart blood deficiency

HT 7, PC 6, CV 14, CV 15, BL 17, BL 20

- Phlegm—fire harassing the Heart

PC 7, CV 17, BL 15, CV 12, ST 40, LR 2, LR 3

Liver Disharmonies

- Constrained Liver qi

LR 3, LR 14, LR 13, PC 6

- Liver blood deficiency

BL 17, BL 18, BL 20, LR 8, ST 36

- Ascendant hyperactivity of Liver yang

LR 2, LR 3

General Disharmonies

- Qi deficiency

GV 20, ST 36, CV 12, CV 4

- Qi deficiency failing to control Blood

GV 20, ST 36, BL 17, CV 4

- Qi deficiency with Blood stasis

GV 20, ST 36, SP 10, BL 17, KI 9, CV 4

- Blood stasis

SP 10, BL 17, KI 9

- Blood heat

SP 10, BL 17, LI 11, LR 2, LR 3, SP 1, KI 2, PC 3

- Trauma

GV 20, CV 12, PC 6

Exterior Disharmonies

- Exterior cold

LU 7, TE 5, GB 20

- Exterior heat

LI 11, TE 5, GV 14, GB 20

Touch Intervention

The active control group will receive touch care in the form of light pressure applied to non-acupuncture points to provide non-specific therapeutic effects.

This touch treatment will consist of light pressure to each of the five points outlined below for four minutes for each point. Treatment consists of two treatments in the first week of entering the trial followed by a weekly visit until participants reach 12 completed weeks of gestation.

Touch is included to account for the non-specific therapeutic treatment effects that can be seen to be present in the acupuncture treatment group, such as attention, care, respite from daily stress, being placed in a relaxing environment, receiving treatment, and spending time with, and being touched by, a treatment provider.

A written sheet detailing dietary and lifestyle risk factors will also be given to women receiving supportive care treatment

Touch Protocol

Each of the following five points will be lightly held for four minutes, giving an overall treatment time of 20 minutes, with the points to be used in the following order.

Point	Patient position	Anatomical landmark	Point location	Relationship to channel
Foot	Supine	Metatarsal bones	Dorsum of the foot. In the hollow distal to the junction of the 3rd and 4th metatarsal bones	Midway between the GB and ST channel, approximately at the level of LR 3
Lower leg	Supine, knee partly flexed	Anterior crest of tibia	On the anterior crest of the tibia, 7 cun below the base of the patella	1 cun distal and 1.5 cun medial to ST 37
Forearm	Supine	Lateral epicondyle of the humerus	On the extensor surface of the forearm	Midway between LI and TH channels one cun distal and one cun lateral to LI 11
Hand	Supine	Metacarpal bones	Dorsum of hand in the hollow distal to the base of the 2nd and 3rd metacarpal bones	Midway between the SJ channel and the extra point Luozhen (M-UE-24), approximately at the level of SI 3
Chest	Supine	Clavicle	Antero lateral aspect of the chest. Inferior border of the clavicle, 5 cun lateral to the midline	Midway between the ST and LU channels—at the level of ST 13 and LU 2
