# Supplementary material for: Does acupuncture have a role in the treatment of threatened miscarriage? Findings from a feasibility randomised trial and semi-structured participant interviews
Source: BMC Pregnancy Childbirth. 2016 Oct 7;16:298. doi: 10.1186/s12884-016-1092-8 (PMC5055689; doi:10.1186/s12884-016-1092-8)
Supplement: Additional file 2: — Diet and Lifestyle sheet. This supplementary file details the medical self-care advice given to the women in this trial. (PDF 199 kb) [file 12884_2016_1092_MOESM2_ESM.pdf]

DIET AND LIFESYLE SHEET FOR THREATENED MISCARRIAGE

The following information represents the best evidence medicine advice currently available and is intended as a guide to assist you in making dietary and lifestyle choices at this time in your pregnancy.

Alcohol: Alcohol crosses placental barrier which means that any alcohol you drink passes through to your baby. At present it is not known if alcohol is safe during pregnancy or how much alcohol you need to drink before it becomes a problem for your baby. The New Zealand Ministry of Health guidelines recommend the safest approach is to choose not to drink at all while pregnant.
http://www.healthed.govt.nz/resources/whenyoudrinksodoesyourbaby.aspx

If you would like further information, are worried about the amount the alcohol you are drinking or unable to stop drinking, you can speak to your doctor or midwife for support and advice or phone **0800 787 797** for a free support helpline.

Bed rest: There is no evidence that being on complete bed rest, either at home or in a hospital reduces your risk of miscarriage.

Caffeine: Caffeine crosses the placental barrier. High levels of caffeine during pregnancy may increase the risk of miscarriage or having a baby with a low birth weight. Although high levels are found in coffee, caffeine can also be found naturally in tea and cola drinks. The recommendation is to reduce the amount of caffeine you drink during pregnancy to a maximum of the 300mg per day.

The following is a list of the average caffeine content that can be found in:

- 1 regular long black coffee 211 mg
- 1 cappuccino 105 mg
- 1 cup plunger coffee 66 mg
- 1 cup of instant style coffee 51 mg
- 1 cup of tea (loose leaves) 57 mg
- 1 cup of tea (tea bag) 47 mg
- 1 regular cola drink 35 mg

Energy drinks may also contain caffeine which has been added. These drinks may also contain other ingredients not recommended for pregnant women.
Swapping regular coffee for decaffeinated varieties which will reduce caffeine intake, however safe levels of decaffeinated products for pregnant women are as yet unknown.

For further information about the caffeine content in food and drink
http://www.foodstandards.gov.au/foodmatters/caffeine/index.cfm

Cigarette smoking: The detrimental effects of cigarette smoking are well documented and show an increased risk factor for miscarriage as well as problems later in pregnancy such as babies that are born prematurely, low birth weight babies and stillbirth.
If you are interested in stopping or reducing your smoking your doctor or midwife can give advice about the options there are to help you while you are pregnant. Further advice is also available at: Quitline http://www.quit.org.nz
or phone **0800 778 778** to speak to a Quitline Advisor for free telephone support.

Exercise: There is no evidence to show a link between exercise in early pregnancy and miscarriage, however if you are have vaginal bleeding it is advised that you seek the medical advice from your doctor or midwife before commencing or continuing with an exercise programme.

Multivitamin supplements: For most women, with the exception of a pregnancy folate supplement, vitamins are not recommended during early pregnancy. There is the concern that supplements not designed for pregnancy may be associated with excessive doses of certain nutrients, such as Vitamin A. A healthily diet is recommended as the best way to obtain the vitamins and minerals required in early pregnancy. However women with vegetarian and vegan diets may require additional supplementation and will need to discuss this with their doctor or midwife. For further information on eating a healthy diet in pregnancy:
http://www.nhmrc.gov.au/publications/synopses/ds10syn.htm

Sexual Intercourse: This is a personal choice for couples as there is no evidence that sexual intercourse increases your risk of a miscarriage.

For further advice about any of the diet and lifestyle information in this leaflet please consult your doctor or midwife.
