# Supplementary material for: Does acupuncture have a role in the treatment of threatened miscarriage? Findings from a feasibility randomised trial and semi-structured participant interviews
Source: BMC Pregnancy Childbirth. 2016 Oct 7;16:298. doi: 10.1186/s12884-016-1092-8 (PMC5055689; doi:10.1186/s12884-016-1092-8)
Supplement: Additional file 3: — Interview Schedule for Trial Participants. This supplementary file details the interview questions for women being interviewed. (PDF 260 kb) [file 12884_2016_1092_MOESM3_ESM.pdf]

Interview Schedule for Trial Participants

Introduction

I have some questions to ask you about the treatment you received as part of the trial you took part in following your diagnosis for threatened miscarriage. Please feel free to bring up any comments as they come into your mind—these questions do not need to be answered in any special order. If you feel uncomfortable with any of the questions, please tell me. I can stop the interview and turn off the recorder at any time.

Questions

1) Can you tell me why you entered into this study?

2) It would be helpful if you could tell about the treatment you received.

A) Were there any specific effects you noticed immediately following the treatment?

B) Any specific effects in the week following the treatment?

C) Did you notice any other changes in your general health—physical or emotional?

D) Do you feel that the treatment you received made you feel any differently about your threatened miscarriage?

E) Have you received any form of acupuncture or touch therapy before?

F) Were there any specific effects you were hoping to notice with the treatment?

3) Tell me about the treatment sessions.

A) Describe what happened in the first session.

B) Looking back on the treatments you received, is there anything from the sessions that especially sticks in your mind?

C) Was there anything that occurred during the treatment that you found difficult?

4) If you had a friend experiencing a threatened miscarriage, what advice would you give your friend about possible treatment?

5) Is there anything else that you would like to say about the treatments you received?
